# Enhancing men's awareness of testicular diseases (E-MAT) feasibility trial: Protocol for a mixed method process evaluation

**DOI:** 10.12688/hrbopenres.13515.2

**Published:** 2023-09-22

**Authors:** Josephine Hegarty, Megan McCarthy, Martin Davoren, Frances Shiely, Janas M. Harrington, Gillian Shorter, David Murphy, Eoghan Cooke, Billy O'Mahony, Mohamad M. Saab

**Affiliations:** 1 School of Nursing and Midwifery, University College Cork, Cork, Ireland; 2 Sexual Health Centre, Cork, Cork, Ireland; 3 Trials Research and Methodologies Unit, Health Research Board Clinical Research Facility, University College Cork, Cork, Ireland; 4 Health Research Board Centre for Health and Diet Research, School of Public Health, University College Cork, Cork, Ireland; 5 Centre for Improving Health Related Quality of Life, School of Psychology, Queen's University Belfast, Belfast, Northern Ireland, UK; 6 School of Computer Science and Information Technology, University College Cork, Cork, Ireland

**Keywords:** Virtual reality, knowledge, athletes, testicular diseases, social media, technology

## Abstract

**Background**: Testicular cancer (TC) is the most common malignancy in men under 50 years. Athletes are particularly at risk of testicular trauma and diseases. Experiencing negative testicular symptoms does not necessarily imply that men seek help. Men’s awareness of testicular diseases is often lacking and their intention to seek help for testicular symptoms is sub-optimal. The use of virtual reality (VR) may be effective in promoting men’s awareness of testicular diseases.

The Enhancing Men's Awareness of Testicular diseases (E-MAT) feasibility trial aims to test the effect of E-MAT _VR _(intervention; interactive experience using virtual reality [VR]) compared to information delivered Electronically E-MAT _E_ (control; same information as E-MAT _VR _delivered as plain text and images) on testicular knowledge, and testicular self-examination among male athletes affiliated with a national sports organisation.

The overall aim of this mixed method process evaluation will be to describe (i) the experiences of participants and key stakeholders (e.g., researchers); (ii) the perceived effectiveness of intervention components; (iii) acceptability of the feasibility trial and intervention procedures; (iv) the relationship between implementation, mechanisms, and context; and (v) the barriers and facilitators to support effective conduct of a future definitive trial.

**Methods: **This mixed method process evaluation will use a descriptive realist evaluation. Quantitative data will be gathered using a usability and satisfaction survey, in addition to fidelity checks during intervention delivery. Quantitative data will be analysed using descriptive and inferential statistics. Qualitative data will be gathered from semi-structured interviews and focus groups with participants and key stakeholders to investigate their experiences of E-MAT _VR_ and E-MAT _E_, and explore areas for improvement. Thematic analysis of transcripts will be conducted.

**Conclusions: **This process evaluation will provide an in-depth understanding of how the interventions worked within this cohort and lessons for a future definitive trial.

## Introduction

Testicular cancer (TC) is the most common malignancy in men under 50 years. TC incidence has doubled internationally over the last 30 to 40 years, primarily affecting Caucasian men in Europe ^1^. In Ireland, the most recent data available revealed that 176 men developed TC (2.4% increase/year) and 7 men died from it in 2018, with 91% of cases and 75% of deaths occurring in men under 50 years ^2^.

TC often has a narrow symptom signature (i.e., single identifiable symptom) with high predictive value. A painless mass is the most frequently reported sign of TC, which is often discovered by men in 80% of cases ^3^. It is estimated that 80% of testicular lumps are benign ^4^. However, as such, it is estimated that approximately 20% of testicular lumps are either precancerous or cancerous ^4^. This suggests that men ought to be educated to practice self-examination to familiarise themselves with what is normal for them and what is not ^5^.

Epididymo-orchitis, a clinical syndrome consisting of inflammation of the epididymis and/or testes ^6^, is the most common cause of testicular lumps, accounting for 1/144 outpatient visits in sexually active men under 50 years in the United States of America (USA) ^4^. If left untreated, epididymo-orchitis can lead to sepsis and infertility ^7^. Testicular torsion affects 1/4,000 men under 25 years and is often associated with trauma such as sports injuries ^8^. Torsion causes scrotal pain and vomiting and can lead to necrosis if testicular blood supply is not restored within six hours from the onset of pain ^9^.

Orchiectomy, a surgical procedure to remove one or both testicles, is the most common treatment modality for TC. This can be followed by chemotherapy and/or radiotherapy, particularly in advanced disease ^10^. The reports of 215 orchidectomies performed over two time periods (1975–1985 and 2007–2012) found a significant reduction in mean tumour size from 7.2 cm in 1975–1985 to 4.1 cm in 2007–2012 ^11^. The trend in reduction of tumour size was supported by the increased awareness of TC in the United Kingdom (UK) ^11^. Treatment for advanced cases of TC can be expensive, with one study finding a 2.4:1 cost benefit ratio for early detected TC versus advanced-stage TC ^12^. The cost of detection, treatment, and follow-up for one advanced TC equated to 180–190 clinic visits, suggesting that promoting TC awareness and self-examination is cost saving ^12^.

Some sports are associated with an increased risk of testicular trauma and subsequent disease. A survey of 731 athletes in the USA found that the prevalence of testicular injuries in field games was 48.5% ^13^. Prompt physical examination and treatment are essential in investigation among athletes who experience testicular injuries, and surgical exploration should be considered in all cases of concerns for testicular survival ^14^. For example, hurling, one of the oldest and fastest Irish field games, is played with a sliotar (a hard solid sphere slightly larger than a tennis ball, consisting of a core covered by two pieces of leather stitched together) and struck with a hurley (a long wooden stick with a curved bas at the end) ^14^. When struck, the ball can reach a speed of 160 km/h which poses significant injury risks either by the ball or the hurley ^15^. A single-centre Irish study reported that, out of 70 patients presenting with penoscrotal injuries, 10 (14%) had injuries caused by blunt scrotal trauma whilst playing hurling ^15^. While testicular trauma and diseases are common in hurling, they also often occur in Gaelic football, which is an Irish field game played with a round football that can be caught, kicked and hand passed. One study examining common testicular injuries among different sports found that, of seven patients who presented to the emergency department with testicular pain and swelling, two of these patients presented due to injuries which arose from Gaelic football ^14^.

Experiencing unpleasant testicular symptoms does not necessarily imply that men would seek help. We previously interviewed 29 men about their intentions to seek help for testicular diseases and found that most men interviewed intended to delay help-seeking, mainly due to lack of awareness and symptom misappraisal ^16^. Therefore, raising awareness, and promoting testicular self-examination and early help-seeking can help detect testicular diseases early, reduce treatment costs, and improve health outcomes.

We previously conducted a pre-post pilot study to enhance men's awareness of testicular diseases, help-seeking intentions for testicular symptoms, and intention and behaviour to feel their testes ^17^. This study involved an intervention which engaged men in a three-level, educational, virtual reality (VR) experience ^17^. The intervention was found to be successful in promoting knowledge, testicular awareness, implementation intentions, help-seeking intentions, and behaviours ^17^.

The use of VR may be effective in promoting men’s awareness of testicular diseases ^17, 18^, but the evidence base to support the use of VR in men’s health promotion is limited. Moreover, to the best of our knowledge, no trials have explored the use of VR in men’s health promotion in the past. In addition, no studies evaluated the process of delivering VR as a part of a trial from the perspective of participants and researchers ^18^. The use of VR may be effective in promoting the wellbeing of hard-to-reach populations, particularly among young men, to help raise awareness and promote testicular self-examination and early help-seeking. Therefore, this can help detect testicular diseases early, which in turn can help reduce treatment costs, and improve overall health outcomes.

### E-MAT feasibility trial

The E-MAT feasibility trial will aim to test the effect of E-MAT _VR_ (Intervention: interactive experience using VR) compared to E-MAT _E_ (Control: electronic information), on testicular knowledge and testicular self-examination behaviours among male athletes. The feasibility trial will take place in six geographically distributed Gaelic Athletic Association (GAA) sports clubs in the counties of Cork and Kerry in the Southwest of Ireland, over a three-month period. For each of the six participating GAA clubs, individual participants will be randomised to either the intervention or control arm. The primary outcomes measured in the feasibility trial are testicular knowledge and testicular self-examination behaviours. A full protocol for the feasibility trial is available on ClinicalTrials.gov ^19^. Given that the goal of a feasibility trial is to identify problems that would impede the conduct of a larger, definitive efficacy trial, we have set the sample size at 59, which is aimed at being able to detect failures in study processes that would occur just 5% of the time (with 95% confidence). Bearing in mind a potential attrition rate of 25%, 74 participants will be recruited into the feasibility trial.

A Study Within A Trial (SWAT) will be conducted alongside the feasibility trial to determine which recruitment method (Twitter, Facebook, or Quick Response [QR] code via a poster) is more efficient and cost effective for recruiting men to this feasibility study. A protocol for the SWAT has been accepted in the Northern Ireland SWAT repository ^20^.

The present paper presents a protocol for a process evaluation of the E-MAT feasibility trial using the Medical Research Council (MRC) Guidance for Process Evaluations.

### Description of the intervention

E-MAT _VR_ is an educational intervention to enhance men’s knowledge of testicular diseases and therefore promoting self-examination and early help-seeking for symptoms of concern ^17^. E-MAT _VR_ is a computer software installed onto VR technology and delivered using a wireless headset, handheld controllers, and voiceover. The voiceover script is based on evidence from our prior research ^17, 21^ and the Preconscious Awareness to Action theoretical framework ^22^. E-MAT _VR_ comprises an interactive gaming experience set in an apartment with three distinct areas and takes approximately 10 minutes to complete. The user is required to complete one level to move to the next level. E-MAT _VR _begins with a popping series of words (like opening movie credits) as the voiceover reads them out. These include light-hearted synonyms for the testes (e.g., balls, nuts, gonads). The word “nuts” rather than “testes” is used to engage men with this intimate/sensitive topic.

E-MAT _VR_ Area 1 involves a 3D space represented as an oversized shower with two walnuts in the centre. The user is asked by the voiceover to move around the walnuts using the handheld controllers, while providing facts about the normal size and shape of the testes. Three changes (lump, swelling, and pain [represented as flashing light]) then appear consecutively. These are accompanied with a humorous emotional response from the voiceover. The user must find all three changes to move to the next area.

During E-MAT _VR_ Area 2, a 3D model of a testis is used. The spermatic cord, epididymis, and tumour are represented in this model. During this level, the voiceover links symptoms experienced in Area 1 to testicular structures. For example, the spermatic cord lights up to indicate testicular torsion and a purple lump appears, indicating a growth/lump. The user must click on all three structure to move to the next area.

**Figure 1.**
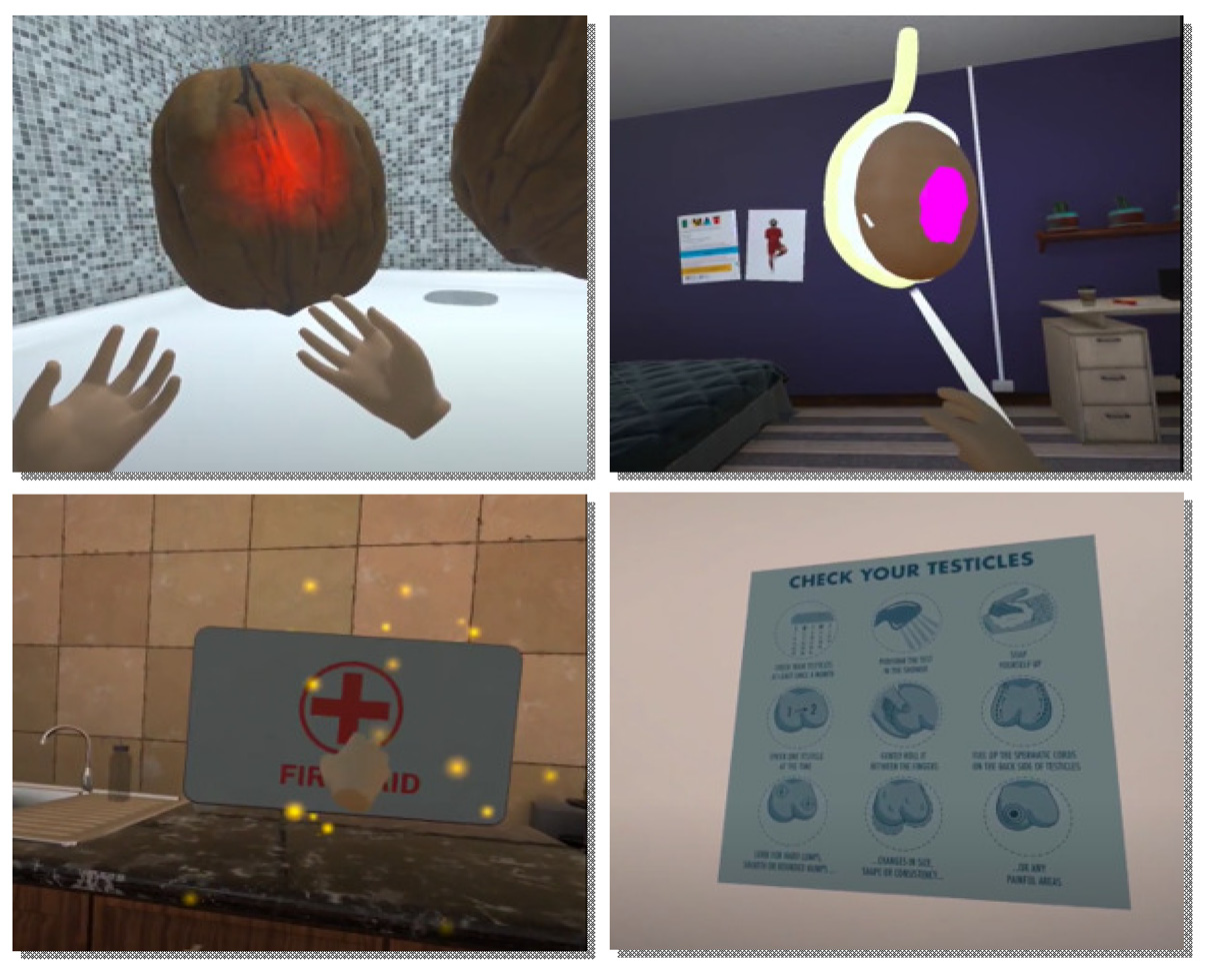
Screenshots from the E-MAT _VR_ intervention.

During E-MAT _VR_ Area 3, key messages are reiterated by the voiceover using three visual representations: (i) a poster featuring a fingerprint to remind participants that their testes are unique and highlight the importance of knowing what is normal for them; (ii) an infographic with the method for testicular self-examination; and (iii) a first aid kit to prompt participants to seek help for abnormalities and seek emergency/immediate attention for severe testicular pain. The screenshots from the E-MAT _VR_ intervention are presented in Figure 1.

E-MAT _E_ serves as the control arm. It involves using the same information as E-MAT _VR_ delivered as plain text (e.g., PDF) with screenshots from E-MAT _VR_. Participants are given 10 minutes to read the text/look at images using a tablet (e.g., iPad).

Interventions will be delivered in person on-site in the individual GAA sports club whilst being cognisant of the public health guidance regarding COVID 19, and infection prevention and control at the time of intervention delivery.

### Aims and objectives

This mixed method process evaluation aims to (i) explore participants’ experiences of E-MAT _VR_ and E-MAT _E _and key stakeholders experiences of delivering the interventions; (ii) investigate the process components including fidelity, dose, and reach; (iii) consider barriers and facilitators that affected implementation, including recommendations to support effective implementation of future trials; and (iv) understand and mitigate potential sources of intervention failure (contextual effects, inputs, engagement, activities, outcomes).

## Methods

### Study design

**Figure 2.**
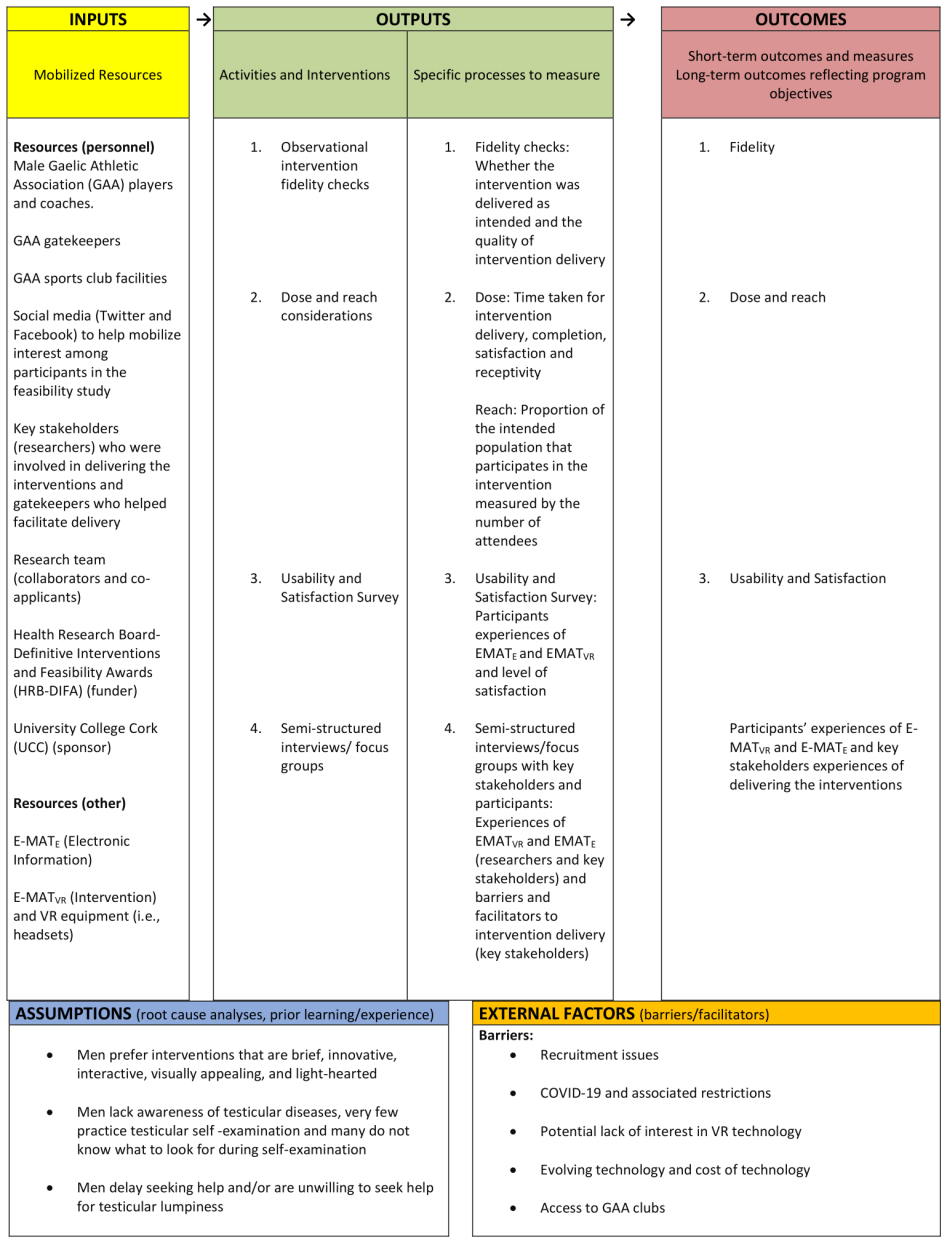
The logic model.

This mixed method process evaluation will apply a descriptive realist evaluation informed by the MRC guidance ^23^ to explore “what works, for whom, under what circumstances” ^24^. To summarise the various elements involved in the intervention, and in keeping with the MRC guidance on process evaluations of complex interventions ^23^, we produced a logic model (see Figure 2), based on the Kellogg Foundation Framework ^25^. The logic model systematically and visually presents and shares our understandings of the relationships among the resources used to underpin the intervention by outlining the inputs, activities, outputs, outcomes, and impact that are anticipated if the intervention proceeds as planned. The underlying assumptions are also described. The logic model will form the basis for ascertaining if the E-MAT _VR _and E-MAT _E_ were implemented as expected and what the potential mechanisms of action were. 

### Study population and setting

Participants will be eligible to participate in this study if they are biological males, members (i.e., players and coaches) of the target GAA clubs, residing in Ireland, and aged 18–50 years (age group at risk for testicular diseases). Participants will be excluded if they are not members of the target GAA clubs. Participants will be also excluded from participating in this study if they have a history of seizures and/or a history of motion sickness. Of note, motion sickness occurs among 20% of VR users and is linked to extreme gaming involving rapid and sudden movements (e.g., riding a roller-coaster and shooting) ^26^. This risk is very minimal with E-MAT _VR_ since the intervention does not involve sudden movements and participants will have full control over their movement throughout the intervention. Moreover, very little evidence exists around the use of VR and seizures. The risk of seizures may also be linked to extreme gaming that involves rapid and sudden movements. This risk is also very minimal in E-MAT _VR_.

### Interviews and/or focus groups

Data will be collected from study participants and key stakeholders. The data collection instruments that will be used are presented in Table 1. While participants fill out and complete a consent form for the feasibility trial, they will also be provided with a separate consent form for the process evaluation. Participants will be invited to participate in either an individual interview or focus group immediately after they complete the intervention in person or virtually (e.g., via Microsoft teams). When this is not possible, an interview at a later date that suits participants will be arranged as soon as possible. This will be to help maximise participation among GAA players and coaches who may not want to disclose their views in a group setting. Due to the sensitive nature of our topic, some participants may feel more comfortable revealing personal and sensitive information in an individual interview. The use of individual interviews and focus groups will help enhance data richness, depth, and transparency ^27^. The interviews and focus groups will explore how E-MAT _VR _and E-MAT _E_ were perceived by participants and key stakeholders, if/why this varied, and how these perceptions affected intervention receptivity. The interviews and focus groups will also explore the mechanisms through which the intervention brings about changes in testicular awareness, which is crucial to understanding both how the effects of the specific intervention occurred and how these effects might be replicated by similar interventions in the future ^24^. There may be some participants purposively selected with varied experiences (for instance we particularly wish to engage with non-completers) to capture the full range of participant perceptions. In the context of our study, non-completers are defined as those who do not complete E-MAT _VR_ or EMAT _E_. Participants who have officially withdrawn from the feasibility study will not be invited.

**Table 1.** Data collection instruments.

Instrument	Purpose	No. of Items *No. of Items in * E-MAT- _VR_ =EMAT- _E_* * unless otherwise* * indicated*	Time administered	Answer options	Scoring	Interpretation of scores
**Usability and** **Satisfaction** **Survey**	To gather the opinions of participants in relation to levels of satisfaction with the interventions and to assess different domains of usability of the interventions	15 items divided as follows: (i) Usability Survey (9 items) (ii) Satisfaction Survey (3 items) (iii) Open-ended questions (3 items)	T1	(i) Items assessed on a 5-point Likert scale (ii) Items assessed on a 5- point Likert Scale (iii) Open-ended questions	(i) Answers range between “Strongly Disagree=1; Disagree=2; Neutral=3”; “Agree=4”; and “Strongly Agree=5” Scores range between 1 and 5 (originally 5-25) (ii) Answers range between ‘Extremely dissatisfied=1’, ‘Dissatisfied=2’, ‘Neither satisfied nor dissatisfied=3’, ‘Somewhat satisfied=4’, ‘Extremely satisfied=5’ (iii) N/A	(i) Higher scores indicate greater usability (ii) Higher scores indicate greater satisfaction (iii) N/A
**Interviews** ** and focus** ** groups**	To explore participants’ experiences of E- MATVR and E-MATE and key stakeholders experiences of delivering the interventions	Participants (7 questions); key stakeholders (10 questions)	T1	Semi-structured interview/focus group guide: Open-ended questions	N/A	N/A
**Intervention** ** Fidelity** ** Checklist**	To check if the intervention was delivered as intended	E-MAT _VR_ (7 items); E-MAT _E_ (3 items)	During intervention	**E-MAT _VR_** 7 items assessed on a 3-point Likert scale	**E-MAT _VR_** (i) Answers range between ‘Complete=1’, ‘Partially complete=2’, ‘Not complete=3’ *(5/7 items)* (ii) Answers range between ‘Yes=1’, ‘Partially=2’, ‘No=3’ *(1/7 items)* (iii) The answer ranges between ‘No=1’ ‘Nausea=2’, ‘Unwellness=3’, ‘Other=4’ *(1/7 items)* **E-MAT _E_** (i) Answers range between ‘Yes=1’. ‘Partially=2’, ‘No=3’ *(2/3 items)* (ii) Time spent on E-MAT _E_ *(1/3 items*	**E-MAT _VR_** (i) and (ii) Lower scores indicate the intervention was done as planned. Higher scores indicate the intervention was not done as planned. (iii) If the VR intervention ended early, the reason is indicated. **E-MAT _E_** (i) Lower scores indicate the intervention was done as planned. Higher scores indicate the intervention was not done as planned. (ii) N/A
**Headset** ** in E-MAT _VR_** ** intervention**	To collect data on the time taken to deliver the E-MAT _VR_ intervention (dose exposure) and the units of the E-MAT _VR_ intervention completed (dose completeness)	2	During intervention	N/A	N/A	N/A

Key stakeholders refer to the research team who were involved in data collection and gatekeepers who were involved in delivering the interventions or facilitating intervention delivery. Stakeholders will be asked to partake in individual interviews and focus groups to glean their perspectives of delivering the interventions. Based on our sample size for the feasibility study, we aim to recruit 20 participants (study participants and key stakeholders) for the interviews and focus groups as part of the process evaluation.

### Context

Context includes anything peripheral to the intervention that may influence its implementation, reach, or effects. Contextual factors can include local or environmental factors which influence implementation of the intervention and the external factors that interact with and influence the implementation of the intervention. Understanding the context is critical to understanding the potential impact of context on the implementation of the interventions. This will be measured in the survey. Moreover, the interview and focus group topic guide will explore in what context the intervention was effective or ineffective, and whether it might be transferrable to other contexts. The usability and satisfaction survey contains a checklist of items. Of those, 3 items from the checklist examine the potential impact of context on the implementation of the interventions. This includes determining (i) whether the intervention is applicable to men across a range of ages (18 to 50 years); (ii) whether the intervention is applicable to men from different ethnic backgrounds; and (iii) if the intervention is applicable to real life (i.e., if the intervention involves using sensory feedbacks (visual, aural) which are applicable to real life ^28^.

### Fidelity

Intervention fidelity checks (see *Extended data* ^29^) will be performed to check if the interventions (i.e., E-MAT _VR_ and E-MAT _E_) are being delivered as intended. During the fidelity checks, the quality of the delivery will also be assessed. A member of the research team will be present at the feasibility trial interventions to observe and record fidelity. The observational schedule will be based upon the standardised training and the intervention delivery manual procedures. Fidelity checks will involve observing participants during testing and recording (tick box) pre-specified items (e.g., participants’ behaviour, interest, and engagement) ^30^. While participants are using E-MAT _VR_, the researcher will perform fidelity checks using an eight-item checklist. Similarly, while participants are using E-MAT _E_, a fidelity checklist of three items will be used by the researcher. The observer will rate items dichotomously (yes, done as planned; or no, not done as planned). The overall percentage score will be scored as an a priori specification of the ideal and minimally acceptable quality marks for delivery with ≥80% scoring high quality, ≥65% moderate quality, ≥50% low quality, and <50% very low quality ^30^. Of note, the same three researchers will be involved in completing the fidelity checklists. Training will be provided by the lead investigator *a priori*.

### Dose

Data will be collected on dose exposure, dose completeness, and dose satisfaction. The time taken to deliver the intervention/control (dose exposure) and the units of intervention/control completed (dose completeness) will be collected. For each participant in the intervention arm (E-MAT _VR_), this data will be recorded in the VR headset and will be extracted from the headset following the intervention for data analysis. The time it takes participants in the control arm (E-MAT _E_) will also be observed and manually recorded. Each participant will be given a unique number which will enable the team to correlate the data from participants in E-MAT _VR_ and E-MAT _E_. Participant’s satisfaction with the intervention, interactions with research staff (dose satisfaction), and extent to which participants are receptive to the interventions (dose exposure) will be collected immediately post-test (T1) through the Usability and Satisfaction Survey (see *Extended data* ^29^). We will incorporate a NASA-TLX to determine mental effort/workload required to use the system ^31^ and the System Usability Scale (SUS) to evaluate usability ^32^.

### Reach

Reach relates to the proportion of the intended population that participates in the interventions which will be measured by the number of participants within each arm. Reach will be defined in terms of the extent to which the intended participants of the intervention actually come into contact with it ^23^. Study reach will be determined by comparing the number of participants who consent to participate to the number of men who received a link to register their interest in participating but did not.

### Usability and satisfaction survey

Study usability and participant satisfaction will be gathered using a modified nine-item usability survey and three-item satisfaction survey ^33, 34^. The questions in this index were previously adapted for E-MAT and to the Irish context ^17^ and are designed to assess different domains of usability. This includes usefulness, ease of use and level of satisfaction with use. The survey will also include four open-ended questions relating to elements of the feasibility study that worked and/or did not work, and any possible changes participants would like to make. Process evaluation data including timing, difficulties experienced with program components, frequency of adverse events, and implementation problems will also be collected during the study. Implementation problems identified during the trial will be categorised as they arise, including the mechanisms by which the issues are addressed.

## Data analysis

### Quantitative data

Quantitative data will be analysed using a statistical analysis software (e.g., R), in consultation with the feasibility trial statistician.

### Qualitative data

Data from the interviews and focus groups with participants and stakeholders will be analysed using qualitative content analysis with predefined themes reflective of the key constructs within the logic model, with an openness to emergent themes ^35, 36^. Content analysis represents a systematic and objective means of describing and quantifying phenomena ^35– 37^. A software (e.g., NVivo) will be used to manage data analysis. Quantitative and qualitative data will be integrated to provide an overall perspective on the process of implementing E-MAT _VR_ and E-MAT _E_.

### Data management

The Health Research Board Clinical Research Facility University College Cork (HRB CRF-UCC) will provide support with FAIRification, storage, and archiving of data in line with data management and stewardship best practice and FAIR (findable, accessible, interoperable, and reusable) data principles ^38^. A study database ( Castor EDC software) will be set up to facilitate data collection and management ^39^. Castor EDC software is a cloud-based clinical data management platform, enabling researchers to easily capture and integrate data ^39^. A data management plan (DMP) will be developed using DMPonline, which is a web-based tool that supports researchers to develop data management and sharing plans ^29^. The DMP will be shared with the HRB to support an Open Access and FAIR approach to downstream data publication.

### Confidentiality and consent

All participants in the feasibility study will sign an informed consent form at the time of recruitment. They will be specifically asked in the consent form (see *Extended data* ^40^) if they consent to partake in an interview/focus group as part of this process evaluation. Similarly, the key stakeholders will be required to complete an informed consent form (see *Extended data* ^40^) prior to the interviews/focus groups.

Participation in the study and participant identifiers will be treated as confidential and no participant identifiable records or results relating to the study will be disclosed to any third party other than the authorized investigators. Personal identifiers will undergo pseudonymisation. An encryption key, held securely away from the data, will be accessible to the project Principal Investigator at site only. Consent will be obtained for all data to be shared publicly, such as data used in the generation of publications arising from the study, and in accordance with the HRB CRF-UCC Standard Operating Procedures. In line with the UCC Code of Research Conduct, the data will be securely held for a minimum of ten years after the project is completed and will then be destroyed following this period.

### Public and patient involvement

Two of the project collaborators are representatives of the target population (members of GAA). Both collaborators were asked to review and comment on the interview topic guide, participant information leaflet, and consent forms for the participants. Based on their recommendations, changes were made to the phrasing and ordering of questions. The input of the public representatives has been critical to the design of the trial.

### Dissemination

The results of this study will be published in an open access peer-reviewed journal. Dissemination will include presenting at public and patient involvement (PPI) conferences (e.g., National Public and Patient Involvement in Research Conference and the PPI Summer School) and targeting key stakeholders (policy makers) with policy briefs and targeted briefing meetings (e.g., Health Service Executive, National Cancer Control Programme, Irish Cancer Society, and the GAA).

### Ethical approval

This study received ethical approval from the Clinical Research Ethics Committee in University College Cork (ECM 4 (e) 16/11/2021 & ECM 3 22/02/2022)

### Study status

Recruitment for this study is projected to begin in Spring 2022.

## Conclusions

This mixed method process evaluation protocol follows best practice guidance and will collect a wide range of quantitative and qualitative data to provide a comprehensive evaluation of the implementation of the E-MAT feasibility trial. This includes consideration of context, structure, process, fidelity, outcomes, and impact. This process evaluation will provide a more in-depth understanding of how the interventions worked and lessons for a future definitive trial aimed to measure intervention efficacy. The impact of such trial on population-incidences of testicular diseases ought to be considered in the future.

## Data Availability

No data are associated with this article. Open Science Framework: Enhancing Men's Awareness of Testicular Diseases (E-MAT) Feasibility Trial: Protocol for a Mixed Method Process Evaluation. https://osf.io/uexpf ^29^. This project contains the following extended data: Appendix 1. Participant Information Leaflets for the process evaluation (participants and researchers) Appendix 2. Consent Forms for the process evaluation (participants and researchers) Appendix 3. Interview Topic Guides (participants and researchers) Appendix 4. Usability and Satisfaction Survey Appendix 5. Fidelity Checklist (E-MAT _VR_ and E-MAT _E_) Data are available under the terms of the Attribution-Non-commercial-NoDerivatives 4.0 International license (CC BY-NC-ND 4.0).
