# Supplementary material for: Enhancing men's awareness of testicular diseases (E-MAT) feasibility trial: Protocol for a mixed method process evaluation
Source: HRB Open Res. 2023 Sep 22;5:25. Originally published 2022 Mar 30. [Version 2] doi: 10.12688/hrbopenres.13515.2 (PMC10450256; doi:10.12688/hrbopenres.13515.2)
Supplement: Peer review history

# Reviewer report: Reviewer response for version 1

**DOI:** 10.21956/hrbopenres.14746.r35219

**Published:** 2023-08-24

**Authors:** Aderivaldo Cabral Dias Filho

**Affiliations:** 1 Hospital de Base do Distrito Federal, Brasília, Federal District, Brazil

The paper describes a feasibility trial for raising awareness of testicular diseases in young men, comparing virtual reality and electronically delivered information. The population of interest consists of athletes, perceived to be at greater risk for testicular trauma, as well as within the age-risk of testicular cancer (and partially of testicular torsion, a disease mostly affecting pubertal boys ^1^).

 The intervention (E-MAT, virtual reality and electronic) is well described and seems to be encompassing and appropriate. The evaluation process (of the intervention) is quite elaborate -- even complex, I dare say.

 Although information retention is considered in the study, mentions to it are left unspecified (participants will be interviewed at a later date when convenient to them). Perhaps later interviews with determined time frames should be included in the protocol to assess information retention decay.

 One key aspect of disease-awareness interventions is its impact on the population-indices of disease parameters within the studied population, e.g. testicular salvage indices in testicular torsion, T stage in testicular cancer. Although one could argue that including such endpoints would warrant another study, I sense that (at least) a suggestion in this sense should be mentioned in the paper.

 Regarding the partial availability of the methods: I acknowledge that the virtual reality tool cannot be included in the paper, but the electronic presentation (the PDF) could be, as one of the appendices.

Is the study design appropriate for the research question?

Yes

Is the rationale for, and objectives of, the study clearly described?

Yes

Are sufficient details of the methods provided to allow replication by others?

Partly

Are the datasets clearly presented in a useable and accessible format?

Not applicable

Reviewer Expertise:

General and Reconstructive Urology, Urological Trauma, Testicular Emergencies, Testicular Torsion.

I confirm that I have read this submission and believe that I have an appropriate level of expertise to confirm that it is of an acceptable scientific standard.

## References

[1] : Testicular volume and Tanner stage: determinant factors for testicular torsion. *Einstein (Sao Paulo)* .2022;20: 10.31744/einstein_journal/2022AO6605 eAO6605 10.31744/einstein_journal/2022AO6605 35476083PMC9000982

# Reviewer report: Reviewer response for version 1

**DOI:** 10.21956/hrbopenres.14746.r35216

**Published:** 2023-08-14

**Authors:** Malene Roland Vils Pedersen

**Affiliations:** 1 University of Southern Denmark, Odense, Denmark

Overall, the study protocol was very well written with an in-depth description of future studies.

 Introduction

 Overall, very well-written introduction with many components of how testicular cancer may affect male health. However, I am not sure TC is accidentally discovered, as the majority of men will seek medical advice if they become aware of a testicular lump.

 It is correct that TC patients can receive chemotherapy and/or radiotherapy after Orchiectomy. However, this will typically occur if the disease has spread to other organs.  

 Consider a gentler description (e.g., athletes are particularly at risk in some sports…) when referring to athletes has a higher risk of testicular trauma.

 The use of VR is a novel way to make men more aware of testicular disease. If it can reduce treatment costs is an interesting perspective. However, I am not sure reducing cost is the goal, perhaps to have a fast-track diagnostic of testicular diseases or diagnose malignant diseases earlier.

 Overall, a well-described intervention description of the E-mat. Inclusion criteria describe men aged 18-50 years as the age group at risk for testicular disease. Notice that the highest risk of testicular cancer such as seminoma and non-seminoma is seen in men aged 15-35 years of age – however other types of cancer are seen in older men. Testicular diseases can be seen in men of all ages. However, it seems an overall good inclusion criterion to limit the inclusion to males up to 50 years when investigating athletes.

 Figure 1 and Table 1 are appreciated for overview.

 Consider having the same person performing the fidelity checks for consistency (While participants are using E-MATVR, the researcher will perform fidelity checks using an eight-item checklist.)

 You may consider performing a power calculation on the arms after piloting the VR study. Recruitment is described to start in spring 2022, normally a study protocol will be reviewed prior to recruitment. The VR, survey, and focus group interviews are described in a manner that is feasible.

 The authors may consider describing the study's endpoints. Overall, an interesting study focusing on awareness of men’s health.

Is the study design appropriate for the research question?

Yes

Is the rationale for, and objectives of, the study clearly described?

Yes

Are sufficient details of the methods provided to allow replication by others?

Partly

Are the datasets clearly presented in a useable and accessible format?

Yes

Reviewer Expertise:

Ultrasound and risk factors for testicular cancer

I confirm that I have read this submission and believe that I have an appropriate level of expertise to confirm that it is of an acceptable scientific standard.
